# Assessment of Technology Usage and Literacy From a Patient Tech Testing Panel in an Adult Primary Care Practice: Cross-Sectional Study

**DOI:** 10.2196/87757

**Published:** 2026-09-11

**Authors:** Antony Nguyen, Tiffany Chinn, Anthony Louie, Eric Chu, Ashwin Vasudevan, Jane Jih

**Affiliations:** 1 Division of General Internal Medicine, University of California, San Francisco, 1545 Divisadero St, San Francisco, CA, 94143, United States, 1 4158857563; 2 Asian American Research Center on Health, San Francisco, CA, United States; 3 Multiethnic Health Equity Research Center, University of California San Francisco, San Francisco, CA, United States

**Keywords:** tech testing panel, primary care, technology literacy, language barriers, digital health

## Abstract

Tech testing panels (TTPs) can assess the usability of patient-facing health technology and provide critical feedback; TTPs should draw upon diverse patient populations by language preference and with a range of technology usage and literacy to ensure that digital health interventions are equitably designed.

## Introduction

Technology innovations are reshaping health care delivery [1]. To support effective and usable technology innovations in clinical practice, tech testing panels (TTPs) are often included. TTPs are structured, team-based spaces where clinicians and patients can test early versions of digital health interventions, identify potential issues, and offer solutions [2].

There is limited literature on TTP composition. TTPs with patient diversity by sociodemographic characteristics including preferred language and technology literacy could support the design of digital clinical innovations that are person-centered, avoiding a one-size-fits-all approach [3]. As part of an urban, academic primary care practice–based project, a patient TTP was created to support the development of an English/Chinese patient-facing mobile app for patient-reported outcomes. We conducted a cross-sectional survey of the TTP to assess technology usage and literacy, as well as differences by preferred survey language.

## Methods

We conducted a cross-sectional survey to assess the technology usage and literacy of a patient TTP within an adult primary care practice in San Francisco, California, with a diverse patient population. This survey was conducted from April to July 2024.

### Ethical Considerations

The University of California, San Francisco, Institutional Review Board approved study procedures (study 20‐32130). All survey respondents provided informed consent. All data were anonymized. Respondents received a US $5 gift card.

### Creation of the TTP

The TTP is a closed cohort developed as part of a practice-based project focused on developing a patient-facing mobile app in English and Chinese. Patients who had a preferred language of English or Chinese and an email address available in the electronic health record received up to 3 in-language email invitations to join the TTP. The TTP was composed of 471 patients, with about 24% (n=115) preferring Chinese.

### Study Procedures

The 24-item survey assessed self-reported sociodemographic characteristics and technology usage and literacy. Technology usage was measured by the 2020 United States Census American Community Survey Questionnaire [4]. We used the full validated Digital Health Care Literacy Scale (DHLS) to assess a broader range of technology skills [5]. All survey questions were translated to traditional Chinese by a bilingual bicultural individual with review and revision by 2 additional bilingual bicultural individuals. Members of the TTP received up to 3 in-language email invitations to complete the online survey.

### Statistical Analysis

Descriptive statistics and bivariate analyses with Pearson *χ*^2^ and independent *t* test were conducted to observe differences between English-preferring and Chinese-preferring survey respondents. We computed the DHLS score based on the sum of 6 questions.

## Results

Of the 471 TTP members, 221 respondents completed the survey, with an overall response rate of 46.9%. The response rate was 53.4% and 27% among English-preferring and Chinese-preferring respondents, respectively. Mean age was 61.6 (SD 14.9) years, with the sample predominantly identifying as women (57.6%) and White (55.3%) (Table 1). About 8% of respondents reported limited English proficiency. About half of respondents reported attaining an advanced degree. About 12% of respondents had Medicaid.

As shown in Table 2, the most reported device used was an Apple iPhone, followed by desktop computer/laptop. Nearly all participants (99.1%) reported access to the internet at home, primarily via cellular data or high-speech internet plans.

**Table 1.** Sociodemographic characteristics of primary care tech testing panel survey respondents.

	Overall sample (N=221)	English (n=190)	Chinese (n=31)
Age (y), mean (SD), range	61.6 (14.9), 29‐23	63.0 (14.4), 29‐93	52.6 (14.6), 30‐74
<65 (n/N, %)	110/216 (50.9)	89/185 (48.1)	21/31 (67.7)
65 or older (n/N, %)	106/216 (49.1)	96/185 (51.9)	10/31 (32.3)
Gender identity, n/N (%)			
Man	90/217 (41.5)	87/186 (46.8)	3/31 (9.7)
Woman	125/217 (57.6)	97/186 (52.2)	28/31 (90.3)
Other	2/217 (0.9)	2/186 (1.1)	0/31 (0.0)
Race and ethnicity, n/N (%)			
Asian	59/217 (27.2)	28/186 (15.1)	31/31 (100.0)
Black	11/217 (5.1)	11/186 (5.9)	0/31 (0.0)
Hispanic/Latina/Latino/Latinx	15/217 (6.9)	15/186 (8.1)	0/31 (0.0)
Middle Eastern/North African	1/217 (0.5)	1/186 (0.5)	0/31 (0.0)
Native Hawaiian/Pacific Islander	1/217 (0.5)	1/186 (0.5)	0/31 (0.0)
White	120/217 (55.3)	120/186 (64.5)	0/31 (0.0)
Multiracial/other	10/217 (4.6)	10/186 (5.4)	0/31 (0.0)
English language proficiency, n/N (%)			
Not well	17/219 (7.8)	0/188 (0.0)	17/31 (54.8)
Very well or well	202/219 (92.2)	188/188 (100.0)	14/31 (45.2)
Highest educational attainment, n/N (%)			
High school diploma/General Educational Development or less	18/218 (8.3)	7/187 (3.7)	11/31 (35.5)
Associate’s degree and/or greater than high school diploma	27/218 (12.4)	22/187 (11.8)	5/31 (16.1)
Bachelor’s degree	61/218 (28.0)	51/187 (27.3)	10/31 (32.3)
Advanced degree	112/218 (51.4)	107/187 (57.2)	5/31 (16.1)
Household income (US $), n/N (%)			
Less than 50,000	43/181 (23.2)	25/158 (15.8)	17/23 (73.9)
50,000‐99,999	24/181 (13.3)	21/158 (13.3)	3/23 (13.0)
100,000‐149,999	24/181 (13.3)	21/158 (13.3)	3/23 (13.0)
150,000‐199,999	28/181 (15.5)	28/158 (17.7)	0/23 (0.0)
200,000 or more	63/181 (34.8)	63/158 (39.9)	0/23 (0.0)
Insurance, n/N (%)^aa^			
Commercial	142/220 (64.5)	131/189 (69.3)	11/31 (35.5)
Medicaid	32/220 (14.5)	23/189 (12.2)	9/31 (29.0)
Medicare	91/220 (41.4)	83/189 (43.9)	8/31 (25.8)
TRICARE/VA	6/220 (2.7)	6/189 (3.2)	0/31 (0.0)
Other	6/220 (2.7)	6/189 (3.2)	0/31 (0.0)
Sexual orientation, n/N (%)			
Straight/heterosexual	190/215 (88.4)	159/184 (86.4)	31/31 (100.0)
Bisexual	6/215 (2.8)	6/184 (3.3)	0/31 (0.0)
Lesbian/gay/homosexual	17/215 (7.9)	17/184 (9.2)	0/31 (0.0)
Other	2/215 (0.9)	2/184 (1.1)	0/31 (0.0)

a “Check all that apply” question.

**Table 2.** Technology usage and literacy of primary care tech testing panel survey respondents.

	Overall sample (N=221)	English (n=190)	Chinese (n=31)	*P* value
Technology usage, n/N (%)				
“Which of the following [devices] do you use regularly?”^a^				
Android smartphone	26/221 (11.8)	26/190 (13.7)	0/31 (0.0)	.03
Android tablet	12/221 (5.4)	12/190 (6.3)	0/31 (0.0)	.23
Apple iPad	110/221 (49.8)	92/190 (48.4)	18/31 (58.1)	.34
Apple iPhone	193/221 (87.3)	162/190 (85.3)	31/31 (100.0)	.02
Desktop or laptop computer	185/221 (83.7)	168/190 (88.4)	17/31 (54.8)	<.001
Mobile phone that is only used for calling and texting	13/221 (5.9)	13/190 (6.8)	0/31 (0.0)	.22
Other	11/221 (5.0)	11/190 (5.8)	0/31 (0.0)	.37
“At the place where you live, do you have access to the internet?”^a^				.74
Yes, by paying a mobile phone company or internet service provider	218/220 (99.1)	187/189 (99.0)	31/31 (100.0)	
Yes, without paying a mobile phone company or internet service provider	2/220 (0.9)	2/189 (1.1)	0/31 (0.0)	
“Do you or any member of your household have access to the internet using a ____ in this household?”^b^				
Cellular data plan for a smartphone or other mobile device	210/216 (97.2)	179/185 (96.8)	31/31 (100.0)	.60
Broadband (high speed) internet service such as cable, fiber optic, or DSL service installed	195/209 (93.3)	175/185 (94.6)	20/24 (83.3)	.06
Satellite internet service installed	17/206 (8.3)	15/182 (8.2)	2/24 (8.3)	>.99
Dial-up internet service installed	8/199 (4.0)	8/177 (4.5)	0/22 (0.0)	.60
Some other service	5/191 (2.6)	5/169 (3.0)	0/22 (0.0)	>.99
Technology literacy, n/N (%)				
“I can install applications/programs (eg, Zoom, Microsoft Office, Google Chrome, etc) on my mobile phone, computer, or another electronic device on my own without asking for help from someone else.”				<.001
Strongly agree	189/221 (85.5)	169/190 (89.0)	20/31 (64.5)	
Somewhat agree	25/221 (11.3)	19/190 (10.0)	6/31 (19.4)	
Neither agree nor disagree	1/221 (0.5)	1/190 (0.5)	0/31 (0.0)	
Somewhat disagree	3/221 (1.4)	1/190 (0.5)	2/31 (6.5)	
Strongly disagree	3/221 (1.4)	0/190 (0.0)	3/31 (9.7)	
“I can use applications/programs (eg, Zoom, Microsoft Office, Google Chrome, etc) on my mobile phone, computer, or another electronic device on my own without asking for help from someone else.”				.01
Strongly agree	184/221 (83.3)	164/190 (86.3)	20/31 (64.5)	
Somewhat agree	31/221 (14.0)	22/190 (11.6)	9/31 (29.0)	
Neither agree nor disagree	2/221 (0.9)	2/190 (1.1)	0/31 (0.0)	
Somewhat disagree	2/221 (0.9)	1/190 (0.5)	1/31 (3.2)	
Strongly disagree	2/221 (0.9)	1/190 (0.5)	1/31 (3.2)	
“I can set up a video chat (eg, Zoom, FaceTime, Skype, etc) using my mobile phone, computer, or another electronic device on my own without asking for help from someone else.”				<.001
Strongly agree	182/221 (82.4)	163/190 (85.8)	19/31 (61.3)	
Somewhat agree	33/221 (14.9)	24/190 (12.6)	9/31 (29.0)	
Neither agree nor disagree	1/221 (0.5)	1/190 (0.5)	0/31 (0.0)	
Somewhat disagree	2/221 (0.9)	2/190 (1.1)	0/31 (0.0)	
Strongly disagree	3/221 (1.4)	0/190 (0.0)	3/31 (9.7)	
“I can solve or figure out how to solve basic technical issues on my own without asking for help from someone else.”				<.001
Strongly agree	126/221 (57.0)	114/190 (60.0)	12/31 (38.7)	
Somewhat agree	77/221 (34.8)	67/190 (35.3)	10/31 (32.3)	
Neither agree nor disagree	9/221 (4.1)	6/190 (3.2)	3/31 (9.7)	
Somewhat disagree	1/221 (0.5)	1/190 (0.5)	0/31 (0.0)	
Strongly disagree	8/221 (3.6)	2/190 (1.1)	6/31 (19.4)	
“If you encounter a technical issue while using your mobile phone, computer, or another electronic device, what do you do first?”				<.001
Try and solve the technical issue myself—without asking someone else for help	178/221 (80.5)	162/190 (85.3)	16/31 (51.6)	
Ask someone for help	34/221 (15.4)	19/190 (10.0)	15/31 (48.4)	
Other	9/221 (4.1)	9/190 (4.7)	0/31 (0.0)	
“How often do you need someone to help you with using your digital devices?”				<.001
Almost always	8/221 (3.6)	1/190 (0.5)	7/31 (22.6)	
About half the time	20/221 (9.1)	13/190 (6.8)	7/31 (22.6)	
Not very often	151/221 (68.3)	136/190 (71.6)	15/31 (48.4)	
Never	42/221 (19.0)	40/190 (21.1)	2/31 (6.5)	
Digital health literacy score^c^, mean (SD), range	20.3 (3.2), 2‐23	20.8 (2.2), 6‐23	17.2 (5.8), 2‐23	<.001

a “Check all that apply” question.

b “Yes/no” question for each category.

c Score based on the sum of 6 questions, with responses receiving the following scores: strongly disagree=0 points, disagree=1 point, neutral=2 points, agree=3 points, and strongly agree=4 points, with a maximum score of 24.

Overall, technology literacy was high: 85.5% of participants strongly agreed that they could install apps independently and 83.3% could use them without help. More than half strongly agreed they could solve technical issues independently. When faced with technical problems, 80.5% reported attempting to resolve the issue themselves before seeking help.

In bivariate analysis by preferred survey language, Chinese-preferring respondents reported lower levels of technology literacy compared to English-preferring counterparts. Fewer Chinese-preferring participants strongly agreed they could install apps (64.5% vs 89%, *P*<.001), use apps independently (64.5% vs 86.3%, *P*=.012), set up video chats (61.3% vs 85.8%, *P*<.001), or solve technical issues on their own (38.7% vs 60%, *P*<.001). Chinese-preferring respondents were also more likely to report frequent need for assistance with digital devices (43.4% vs 10%, *P*<.001). The DHLS mean score differed by language (Chinese-preferring 17.2, SD 5.8 vs English-preferring 20.8, SD 2.2; *P*<.001).

## Discussion

This overall sample of TTP respondents from a primary care practice reported high technology literacy. However, in stratified analysis by preferred survey language, Chinese-preferring respondents reported lower levels of technology literacy than English counterparts.

Consistent with prior literature, our results suggest that linguistically diverse patients experience technology literacy barriers [6] and reinforce the importance of addressing technology literacy across linguistically diverse groups in the United States to promote accessible health technology design and use [7]. Our study adds new information about TTP composition as TTP composition matters for equitable technology tool development.

This study has a modest sample size, including a small sample size of Chinese-preferring respondents. This study also has limited generalizability due to the study sample being from one primary care practice in one geographic location and due to the study sample being drawn from a TTP composed of self-selected digitally inclined patients with high socioeconomic status and educational attainment who were recruited by email and opted to join the TTP. Nonresponse bias between English and Chinese survey recipients may exist, and the survey was only available online. Our findings are descriptive associations and future studies with larger sample sizes for multivariate analysis are needed. Lastly, self-reported measures may have led to social desirability bias.

Our study of a primary care patient TTP found a difference in technology literacy by preferred language. Recruiting patient TTPs with diversity by language and technology literacy could help support the development of digital health interventions that are more equitable and usable by a broader population.

## Abbreviations

TTP: tech testing panel
DHLS: Digital Health Care Literacy Scale
